# Acetylcholine and choline in honey bee (*Apis mellifera*) worker brood food are seasonal and age-dependent

**DOI:** 10.1038/s41598-024-68650-x

**Published:** 2024-08-06

**Authors:** Paul Siefert, Helene Lau, Vivien Leutz, Sara Diana Leonhardt, Gaby Schneider, Jochen Klein, Bernd Grünewald

**Affiliations:** 1 Institut für Bienenkunde, Polytechnische Gesellschaft, Goethe University, Frankfurt Am Main, Germany; 2 Institute of Pharmacology and Clinical Pharmacy, College of Pharmacy, Goethe University, Frankfurt Am Main, Germany; 3 Plant-Insect Interactions, TUM School of Life Science Systems, Technical University of Munich, Freising, Germany; 4 Institute of Mathematics, Goethe University, Frankfurt Am Main, Germany

**Keywords:** ACh, Nursing, Brood food, Larval development, Nutrition, Seasonality, Developmental biology, Ecology, Physiology, Social behaviour

## Abstract

Nursing honeybees produce brood food with millimolar concentrations of acetylcholine (ACh), which is synthesized through head gland secretions mixed with honey stomach contents. While we previously demonstrated the necessity of ACh for proper larval development, the dynamics of ACh levels throughout ontogenesis and their seasonal variations have remained unclear until now. Our HPLC analysis reveals dependencies of choline and ACh levels on larval development days (LDDs), influenced by seasonal (April–September) variations. Median ACh concentrations peak on LDD 2, declining significantly toward cell capping, while choline levels are lowest during the initial LDDs, rising markedly toward cell capping. Seasonal patterns show peak ACh levels from April to June and a low in August, paralleling choline's peak in July and low in August. This seasonality holds consistently across multiple years (2020–2022) and colonies, despite potential variations in colony performance and environmental conditions. Our analysis found no correlation between temperature, sunshine, precipitation, or favourable foraging days and ACh/choline levels, suggesting the involvement of additional factors. These findings underscore the seasonal fluctuation of ACh levels and its potential implications for the genetic programs governing winter bee development.

## Introduction

Insects have evolved adaptive strategies over evolutionary time scales, including modifications in behaviour, reproduction, and seasonal activities in response to environmental changes^1,2^. Seasonal variations, influenced by latitude, temperature, day length, and precipitation, bring about periodic changes in floral resources, crucial for most insect species^3^. Honey bee colonies undergo annual cycles, involving spring brood rearing, summer growth, and fall resource storage for winter preparation^4^. Foraging on plants, they rely on nectar as energy source, and pollen for essential micro- and macronutrients^5,6^. The foragers adjust their choices based on the colony’s needs and seasonal micronutrient preferences^7–9^, exhibiting variations in their microbiome, potentially linked to food nutritional quality^10,11^. Brood area changes may be influenced by nutrient variations in seasonal pollen, potentially including higher levels of amino acids and fatty acids that contribute to gland growth and brood care in spring pollen^12–18^. Collected pollen is stored in cells and enhanced in stability using worker secretions, potentially increasing its nutritional value^19–21^. Compared to fresh pollen, the stored pollen has less complex polysaccharides (e.g. absence of starch), elevated simple sugars, and altered profiles of amino acids, proteins, and lipids^19,22–24^.

Nurse bees consume the pollen, stimulating the development and protein content of larval food-related glands, primarily the hypopharyngeal gland, and the mandibular gland^25–28^. Gland secretions are highly adapted to the larval lifestyle, easily digestible, and fully utilized, crucial for bee larvae with a blind-ended midgut^29^. Studies identify water, sugar, carbohydrates, proteins, lipids, vitamins, and minerals as key components in larval food for worker and queen bees^30,31^. Nurse bees create diverse larval foods by blending gland products with honey stomach contents, adjusting based on age and caste, for example from "worker jelly" to "modified worker jelly"^32–37^. Differences in the larval food contribute to honey bee caste differentiation, influencing epigenetic regulation and gene expression^33,38–43^. Food composition is thought to impact winter bee generation, affecting various physiological changes^44–47^. Winter bee onset is currently linked to reduced fall pollen availability, with other triggers less clear^48^. One potential trigger molecule in honey bee larval diet is acetylcholine (ACh), found in millimolar concentrations in developing larvae's food^49–52^.

The cholinergic system of insects exhibits notably elevated levels of ACh, acetylcholine esterase, and choline acetyltransferase (ChAT) compared to vertebrates^53^. Only three studies have explored ACh concentration in *Apis* brood food^49,50,54^. Early observations in *Apis mellifera* suggest varying ACh content in worker nutrition for larvae below 5 mg (8.3 mM), between 10–20 mg (5.5 mM), and older larvae receiving modified worker jelly (1.2 mM)^49^. A recent semi-field study reports 4.13 mM ACh in worker jelly for larger larvae, showing the necessity of ACh for honey bee larval development for the first time^51^. The significance of ACh in honeybee larval development has been demonstrated by impairing cholinergic pathways in in-vitro larval rearing^55–57^ or within-colony experiments^58,59^, leading to delayed development and increased larval mortality (review: ^60^). However, a detailed age- and season-dependent analysis of ACh and choline in brood food is lacking to date.

ACh synthesis in hypopharyngeal canal cells, by membrane-bound choline acetyltransferase (ChAT), occurs in an acidic brood food environment (pH ~ 4.0), contributing to ACh stability even after boiling for 2 h^50,51^. In insects, two acetylcholine receptor types, nicotinic (nAChR) and muscarinic (mAChR), were identified (review:^60^). The honey bee nAChR, a cys-loop receptor family member, functions as a cation-selective channel, mediating membrane depolarization and calcium influx upon activation^61–64^. Less studied, insects’ mAChRs include two genetically identified receptors with distinct sensitivities to muscarine, activation by ACh, and varying responses to atropine and scopolamine^65,66^.

Despite the crucial role of ACh in worker development, limited research has explored seasonal variations in ACh within brood food. In this study, we collected brood food samples in April 2018 and from April to September 2020–2022, measuring ACh and choline levels via HPLC throughout worker larval development. Given the documented variability in brood food composition among nurses, we anticipated strong dependencies on the various larval development days (LDDs) for workers. While literature suggests seasonal variations in nutritional composition, we expected ACh and choline levels in brood food to remain relatively stable, considering their crucial roles in larval development. If seasonal variations were to occur, we anticipated yearly differences due to variations in temperature, precipitation, colony development and other factors.

## Methods

### Honey bee colonies

We employed one to two-story Zander format honey bee hives from April to September, situated in the garden of the Institut für Bienenkunde Oberursel (50°13.1’N, 8°32.9’E). The queens were siblings from our local breeding line of the subspecies *Apis mellifera carnica*. Colonies underwent *Varroa destructor* treatment, using 15% formic acid evaporation in early August 2020 and 2021, and complete brood removal in early September 2022.

### Sample extraction and preparation

Brood frames containing all larval instars were extracted from the hives and placed on a heating plate set to 34 °C, with a wet paper towel or cloth on top. This setup prevented hypothermia for the brood, minimizing hive losses, and protected the brood food from drying out due to water evaporation. To accommodate the comb's angular architecture, frames were positioned at a slight angle using a ~ 2 cm high object on the heating plate. A cold light source (KL 1500 electronic, Schott AG, Mainz) facilitated search and extraction.

Each worker jelly sample was obtained from a single cell using a new glass capillary (GC100F-10, Harvard Apparatus, Holliston, USA) carefully placed at the cell bottom for a few seconds to extract worker jelly via capillary forces. A target of 1 mg was aimed for uniformity in subsequent HPLC analysis, determined by the length of the sample in the capillary. Potential risks during sampling included capillary blockage due to misplacement or wax penetration and larval harm. Samples were discarded if larvae were punctured. Additionally, for improved yield, small larvae in cells intended for sampling were gently pushed aside.

We used labelled 1.5 ml reaction tubes that were pre-weighed using an ultra-micro scale with 0.001 mg accuracy (Cubis MSX Sartorius AG, Göttingen), located at the Institute for Ecology and Evolutionary Biology at the University of Frankfurt am Main. The high precision was essential for accurate HPLC results. The worker jelly was then injected into the reaction tubes using a capillary holder (micropipetter, Hirschmann Laborgeräte, Eberstadt), or initially, via a syringe with an adapter fashioned from a silicon tube, a pipet tip, and tube reducing pieces. Samples were promptly placed on ice in a dark Styrofoam box to minimize light-sensitive ACh hydrolysis. After each frame had been fully sampled, samples were stored at − 80 °C until further analysis. Thawed to room temperature, samples were weighed (wet mass) using the ultra-micro scale (Cubis MSX Sartorius AG, Göttingen) in low-light conditions. Complete thawing prevented condensation on the tubes, ensuring stable weight results. Samples were stored between 3 to 501 days at − 40 °C until HPLC analysis due to inconsistent availability (time constraints, over-booking and maintenance). Despite literature suggesting high ACh stability in brood food due to impaired acetylcholinesterase activity due to low pH (see Introduction), we conducted a test (see Supplementary Fig. S1).

### HPLC analysis.

Samples were thawed on ice and 500–1000 µl of water (ROTISOLV®, Carl Roth, Karlsruhe) was added. Samples were vortexed and left for a 15-min resting period; subsequently, samples were vortexed again and centrifuged at 16,000 g for 5 min at 4 °C. 10 µl of the supernatant were transferred to a new tube, 1000 µl HPLC water was added and the mixture was vortexed. 20 µl of the solution were pipetted into a 250 µl microvial (Carl Roth, Karlsruhe), capped with caps for 7 mm microvials (Gilson, Berlin) and briefly centrifuged to remove air bubbles. Samples were injected into the HPLC with the help of an autosampler (SIL-20AC, Shimadzu, Duisburg) set at 7 °C. HPLC analysis was run on an HTEC-500 (Eicom Corp., Kyoto, Japan) using an AC-Gel column (2.0 × 150 mm, reverse phase on polymer) and an enzyme reactor AC-ENZYM II (1.0 × 4.0 mm). The electrochemical detector (ECD) included with the Eicom HTEC-500, with a platinum electrode operating at + 0.45 V, was used for detection. The data was evaluated using eDAQ PowerChrom version 2.7.12. ACh and choline concentrations were determined as nmol/mg and multiplied by the density of royal jelly (1.1 g/ml,^67^) to obtain mM values (1 mM = 0.909 nmol/mg).

### Analysis by larval development time in April 2018.

To minimize disturbances to colonies, we distributed sampling across eight colonies. Following the optimal time schedule for queen confinement, egg marking, and brood food sampling during waking hours, we examined one colony four times at intervals of 24, 48, and 48 h; conducted three sessions with five colonies, each at 48-h intervals; and two sessions with two colonies, also at 48-h intervals. We sampled the brood food at 23 time points throughout larval development, from 0 to 132 h, in 6 h intervals. Extractions occurred from April 12th to April 21st 2018, representing the astronomical spring in the northern hemisphere. For sampling, the queen of each of the eight colonies was confined to an empty comb using a metal comb cage. After a 4-h interval, transparent foil was affixed to the comb to mark cells with eggs. We waited 74 h for the larva to hatch, plus the hours of the time point (0 to 132), and extracted brood food without removing the larvae. Cells were only sampled once. Given the logistical challenges and time-intensive nature of this approach, we transitioned to correlate larval weight with age in the analytical process.

### Analysis by larval weight from April to September 2020–2022.

To monitor seasonal variations in ACh and choline concentrations, we associated larval weight with ACh and choline measurements. Each sample comprised two components weighed individually: the larvae to determine age and the brood food to ascertain ACh and choline concentrations through HPLC analysis. We extracted the larva from the cell using a Swiss larval grafting tool. For large five-day-old larvae occupying the entire cell and proving difficult to extract without damage, we first displaced the surrounding wax of the cell and then removed the larva before capillary insertion. For all other larvae, brood food samples were taken while the larva was still in the cell. Each larva was placed and turned multiple times on tissue to eliminate remaining food, and positioned in a pre-weighed reaction tube or directly weighted (wet mass) on the scale with an accuracy of 0.1 mg (A 120 S, Sartorius AG, Göttingen). Brood food was weighed as previously described.

We analysed a total of 1276 samples from thirteen colonies, each with a unique colony identification number (Table 1). However, due to regular queen replacements in accordance with standard beekeeping practices throughout the experiments, the colony identification number may not consistently align with the same “colony” due to variations in the paternal genetic background.

**Table 1:** Samples obtained by colonies in the seasonal experiments.

Year	Month	Day	Stored	Sample size from colonies by identification number													n_C_	n_S_
				121	243	60	145	186	147	78	111	30	63	68	168	142		
2020	Apr	20	10	15					15	15		15	15				5	75
	May	18	5	15		15			15	15		15					5	75
	Jun	24	3	15		15			15	15		15					5	75
	Jul	20	31	13					15	15			15			15	5	73
	Aug	24	169	15		12			15	15							4	57
	Sep	21	143	14		15			14	15		15					5	73
2021	Apr	22–23	50	14	15	15	13	15									5	72
	May	26	51	15	15	15	15	15									5	75
	Jun	25–26	501	15	14	15		15									4	59
	Jul	21	337	15	15	15	15	15									5	75
	Aug	25	310	15	15	15	14	14									5	73
	Sep	22	292	15	15	15	15	14									5	74
2022	Apr	29	60	15	15		14				15			15			5	74
	May	27	158	15	14		15				11			15			5	70
	Jun	22	23	16	15	15	15				15						5	76
	Jul	20	129	15	15	13	17				16						5	76
	Aug	23	108	12	15	13	12	9			15						6	76
	Sep	21	77	15	16										18		3	49

The sampling year, month, and day are linked to the brood food component's maximal storage duration (in days at − 40 °C), the sample size per colony identification number, and the total number of colonies (n_C_) and sample size (n_S_) per month. Due to regular queen replacements in accordance with standard beekeeping practices colony numbers may not consistently correspond to the same paternal genetic background.

We collected samples from the 18th to the 29th of each month, from April to September in three years from 2020 to 2022. At two of the 18 sampling time points, the sampling required two consecutive days (see Table 1). We aimed to obtain three samples per instar and colony, totalling a maximum of 75 samples per month. Typically, we utilized five hives, but in three out of the eighteen months (Aug. 2020, Jun. 2021, Sep. 2022) we were unable to achieve the targeted samples in the colonies due to missing brood, resulting in the use of fewer colonies. In August 2022 we compensated the lack of brood by introducing an additional sixth hive.

We aimed to cover the five larval development days (LDDs) by comparing weight values to^68^. Since larvae were weighed after brood food sampling was completed, we ended up with relatively few samples for LDD 1 and many for LDD 2 (see Table 2). We included 5 larvae with negative weight as LDD 1 larvae. LDD 5 samples were relatively scarce due to the challenges in estimating their developmental state and obtaining larval food on some sampling dates. We included sixteen larvae weighing above 157.6 mg as LDD 5 larvae for statistical analysis, with weights up to 166.7 mg or 5.7% above the literature value (mean ± SE = 161.54 ± 0.76 mg). Few outliers with larval weights above 170 mg were eliminated.

### Statistical analysis

From a total of 1276 samples we removed eight extreme choline outliers above 9.7 mM, setting the upper limit at the highest value across LDDs of three times the interquartile range above the third percentile (upper limits: 6.0, 5.2, 5.4, 5.8, and 9.7 mM for LDDs 1–5, respectively). In 24 instances, the ACh value was below the HPLC detection limit of 5 fmol/10 µl (1, 1, 4, and 18 cases for LDD 2 – 5, respectively), and these were excluded from the analysis. Sample sizes across LDDs are shown in Table 2. Data was visualized using RStudio 2023.06.2 + 561 for Windows. For trend capture in our April 2018 dataset, we employed locally estimated scatterplot smoothing (loess) with standard error, a non-parametric regression technique combining multiple models based on k-nearest neighbours. Boxplots depict the median, interquartile range (IQR), and outliers, while whiskers extend to a maximum of 1.5 times the IQR from the box. Outliers beyond this range are individually marked for clarity in identifying data distribution characteristics. For stability experiments, we employed a general linear model (GLM) to investigate ACh hydrolysation during storage times. Statistical analyses were performed using IBM SPSS Statistics version 27 with a significance level of 0.05. For simplification of our assumptions, the scale variables ACh (mM) and choline (mM) were initially tested against the ordinal categories of LDD and month (either LDD or LDD per month). Normality was assessed using the Shapiro–Wilk test before applying the Kruskal–Wallis Test for independent samples, followed by pairwise comparisons. Repeated measure testing was not employed, given that colonies underwent individually timed queen replacements as per regular beekeeping practices over the years. Bonferroni correction was applied to adjust significance values for multiple tests, automatically scaling with the number of comparisons (e.g., significance value increased from 0.027 to 0.267 after ten possible pairwise comparisons for five LDD). To take variations of years and colonies into account, we combined all factors in a generalized linear mixed model (GLMM), including colony and year as random factors and LDD and month as fixed factors.

### Ethical approval

This work did not require ethical approval from a human subject or animal welfare committee.

## Results

### ACh and choline dynamics throughout larval development

**Figure 1:**
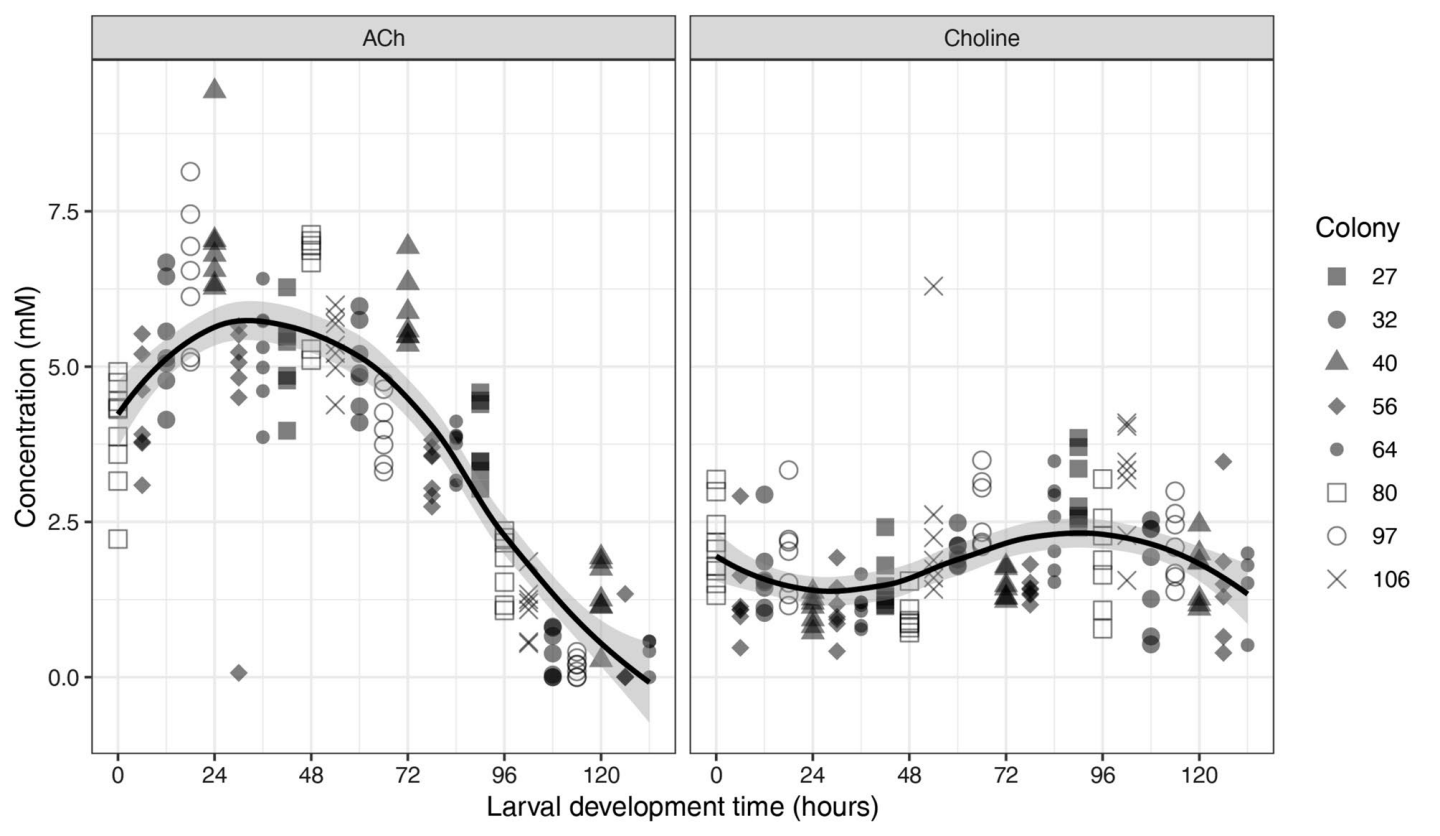
ACh and choline levels in worker food of eight different colonies (displayed by different symbols) in 6-h intervals throughout larval development in April 2018. ACh levels peaked at 5.7 mM at 30 h, followed by a subsequent decline, while mean choline levels remained relatively stable, ranging from 1.7 to 2.3 mM.

In April 2018, we analysed choline and ACh based on larval development time in 6-h intervals. After locally estimated scatterplot smoothing and grouping into 24-h intervals, the mean ACh levels were 4.9, 5.7, 5.2, 3.7, 1.6, and 0.2 mM after 0–120 h, respectively (Fig. 1). The highest concentration of 5.74 mM occurred after 30 h into worker development (on the second development day) and subsequently declined, indicating alterations in worker brood food composition throughout larval development. Despite unevenly distributed 159 data points, discernible differences in ACh levels among the eight colonies were observed. Specifically, 95.2 percent of values from colony 40 exceeded the smoothed mean, while 88.9 percent in colony 56 fell below, indicating performance variations among sister-queen colonies in the same environment. In contrast, mean choline levels remained relatively stable: 1.7, 1.4, 1.8, 2.3, 2.2, and 1.6 mM, respectively, after 0–120 h of larval development (in 24-h intervals).

### Choline and ACh levels across larval development days.

To ensure comparability, we grouped the April to September 2020–2022 data into five larval development days (LDDs) based on weight (see Table 2). Initially, we simplified the assumptions of the determining factor of choline and ACh levels to only the larval development day. Afterwards, we included the month into our analysis to examine the seasonal influences. Finally, we combined all factors in a generalized linear mixed model (GLMM), including colony and year as random factors, to verify our findings.

**Figure 2:**
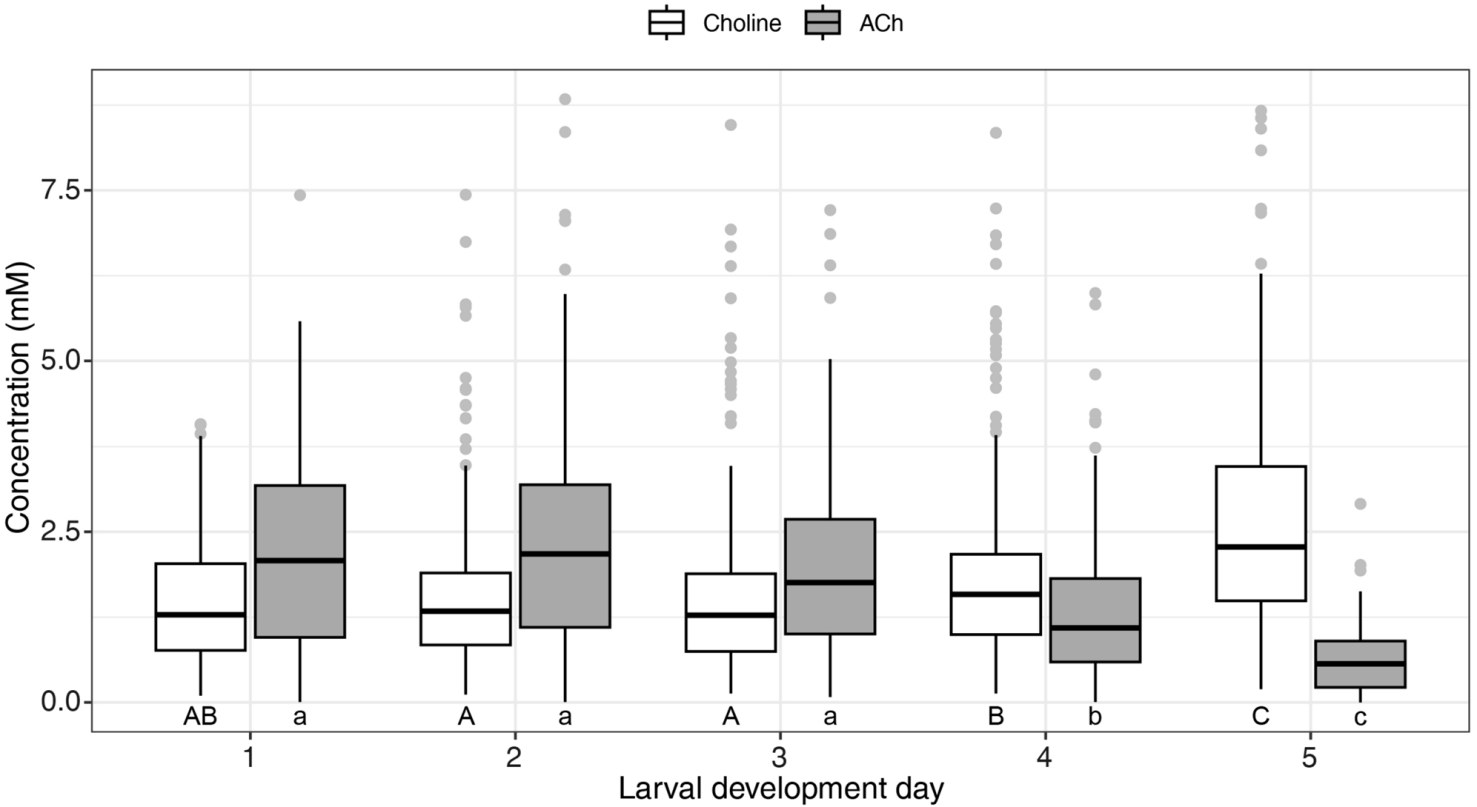
Choline and ACh levels in worker brood food throughout five larval development days pooled over season and year. Median ACh levels peaked on LDD 2, gradually declining towards cell capping, with significant differences (lowercase letters) between LDD 4 and 5 compared to other days (*p* < .001). Median choline levels increased towards cell capping, with significant differences (uppercase letters) on LDD 4 and 5 compared to other days (*p* < .05), except LDD 1 and 4. Sample sizes are presented in Table 2.

Irrespective of month, year and colony, the median ACh levels in worker brood food were 2.0, 2.2, 1.8, 1.1, and 0.6 mM on LDD 1–5, respectively (Fig. 2 and Table 2). This corresponds to 90.9%, 100.0%, 81.8%, 50.0%, and 27.3% of the highest level, indicating a peak on LDD 2 and the lowest level towards the capping of the cell. The larval development day significantly influenced ACh levels (Chi-square(4) = 258.96, *p* < 0.001). Pairwise comparisons indicated no significant difference between LDD 1, 2, and 3, while LDD 4 and 5 were significantly different from all other days (*p* < 0.001 for all comparisons).

**Table 2:** Median choline and ACh concentrations and interquartile range (IQR) in worker jelly samples (n) across larval development days (LDDs) as determined by larval weight.

Larvae			Choline (mM)			ACh (mM)		
LDD	Weight (mg)	n	Median	IQR	n_CH_	Median	IQR	n_AC_
1	0–0.65	137	1.28	1.3	136	2.01	2.3	137
2	0.65–4.69	361	1.34	1.1	358	2.21	2.2	360
3	4.69–24.64	302	1.30	1.1	300	1.76	1.7	301
4	24.64–94.69	301	1.58	1.2	300	1.09	1.3	297
5	94.69–157.64 +	175	2.31	2.0	174	0.57	0.7	157
Total:		1276			1268			1252

Reference weights were used from^68^.

The median choline levels were 1.3, 1.3, 1.3, 1.6, and 2.4 mM on LDD 1–5, respectively (Fig. 2 and Table 2). This corresponds to 54.2% on the first three LDDs and 66.7% and 100% on LDD 4 and 5, respectively, showing increasing levels towards the capping of the cell. The larval development day also had a significant influence on choline levels (Chi-square(4) = 99.24, *p* < 0.001). Similarly to ACh, choline levels did not differ on LDD 1–3. However, LDD 4 and 5 were significantly different from all other LDDs (*p* < 0.05 for all comparisons), except the individual comparison of LDD 1 and 4.

The choline-to-ACh ratio was 0.65, 0.59, 0.72, 1.45, and 4.00 on LDD 1–5, respectively. This shows that on LDD 1–3 choline levels were at approximately 60–70% of the ACh levels before increasing to 145% and 400% on LDD 4 and 5, respectively. The combined concentration of ACh and choline was 3.3, 3.5, 3.1, 2.7, and 3 mM on LDD 1–5, respectively.

### Seasonal impact on ACh and choline levels.

**Figure 3:**
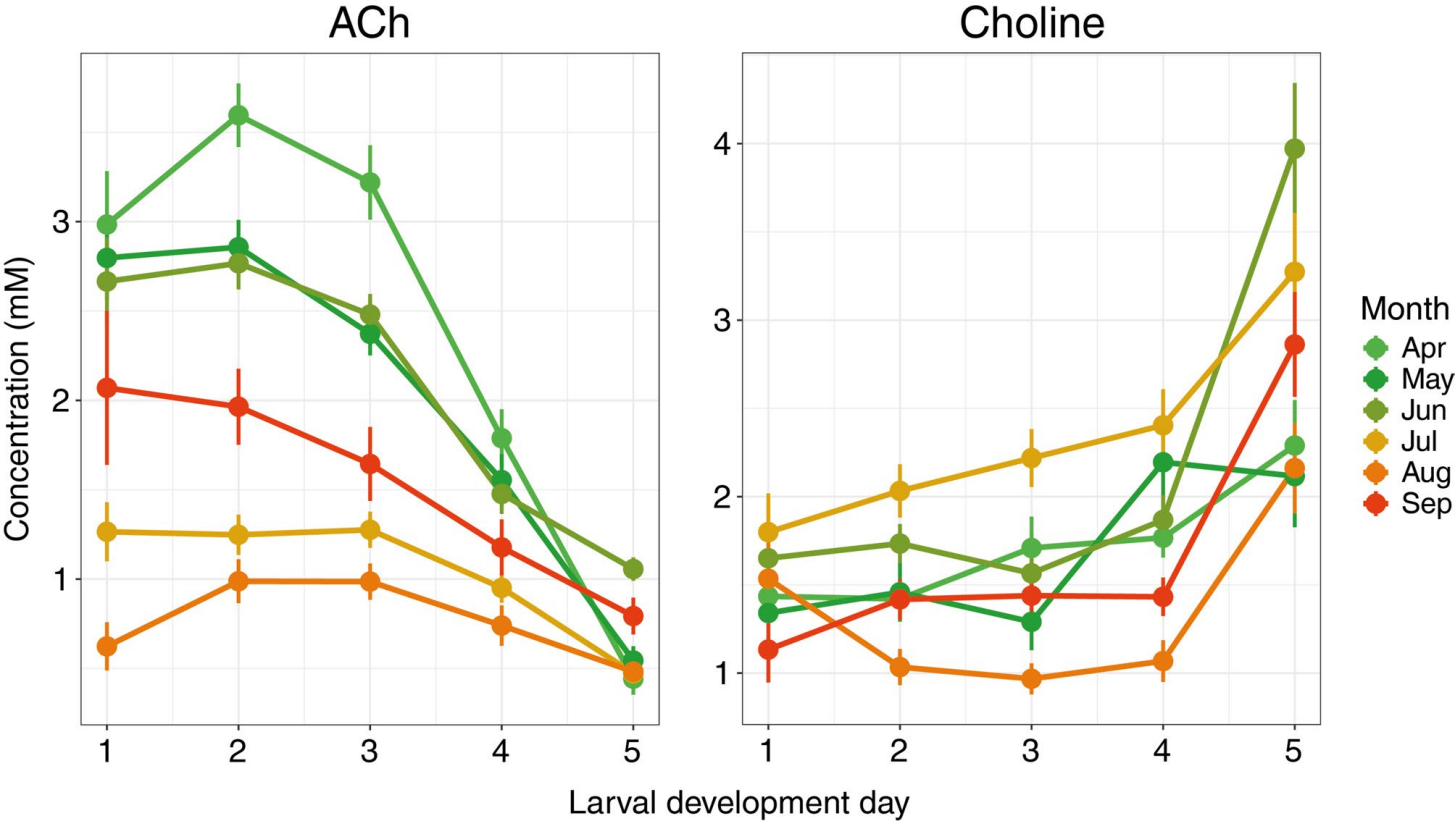
Seasonal influence on ACh and choline levels in worker brood food throughout five larval development days (LDD). ACh levels peaked for 1–4 day-old brood in April, declining steadily throughout the season and reaching the low in August. However, they rebounded in September to levels between those seen in June and July. The most pronounced decrease in ACh levels from young to old larvae was observed from April to June, with August showing the least decline. Conversely, choline levels were notably elevated on LDD 5 compared to LDD 1–3 in all months except August, where they were at their lowest. For visual accessibility the mean and standard error data of the years 2020–2022 was used for this line graph, the statistical analysis was conducted using non-parametric tests (see text). Total sample sizes per LDD are presented in Table 2.

We investigated the effect of the season on ACh and choline levels throughout the five LDDs, by splitting the data per sampling month. The sampling month significantly affected ACh levels on LDDs 1–5 (respective Chi-square(5) and significance values: 51.40, *p* < 0.001; 151.34, *p* < 0.001; 122.98, *p* < 0.001; 44.76, *p* < 0.001; 31.15, *p* < 0.001) and choline levels on LDDs 2–5 (respective Chi-square(5) and significance values: 47.28, *p* < 0.001; 61.38, *p* < 0.001; 45.04, *p* < 0.001; 26.54, *p* < 0.001). As depicted in Table 3, the highest choline median was observed on most LDDs in July, followed by a sharp decline in August. In contrast, the sharp decline in median ACh levels occurred in July already, after elevated levels from April to June, with a less pronounced drop on LDD 4 and 5. Figure 3 presents a visually accessible line graph, depicting mean and standard error data, although the statistical analysis was conducted using the median.

**Table 3:** Median ACh and choline (Cho) concentrations (mM) in worker jelly across larval development days (LDDs) throughout the season (April–September 2020–2022).

LDD	Apr		May		Jun		Jul		Aug		Sep	
	ACh	Cho	ACh	Cho	ACh	Cho	ACh	Cho	ACh	Cho	ACh	Cho
1	2.9	1.4	2.5	1.2	2.9	1.6	1.1	1.7	0.6	1.0	1.5	1.0
2	3.2	1.4	2.7	1.1	2.5	1.6	1.1	1.8	0.8	0.9	1.5	1.3
3	3.1	1.6	2.4	0.9	2.4	1.4	1.1	1.9	0.9	0.8	1.3	1.2
4	1.7	1.6	1.5	1.5	1.3	1.7	0.9	1.8	0.4	0.8	0.9	1.2
5	0.2	2.1	0.5	1.7	1.0	3.5	0.4	3.0	0.5	2.1	0.6	2.6

The larval development day significantly influenced ACh levels in all months from April to September (respective Chi-square(4) and significance values: 91.51, *p* < 0.001; 72.92, *p* < 0.001; 74.69, *p* < 0.001; 31.06, *p* < 0.001; 14.72, *p* < 0.01; 27.54, *p* < 0.001). In April, May and June, ACh levels on LDD 1–3 did not differ but at LDD 4 and 5 they were different from all other LDDs (April:* p* < 0.01 for all comparisons; May: *p* < 0.05 between LDD 4 and 5, and *p* < 0.01 for all other comparisons; June: *p* < 0.001 for all comparisons). In July, ACh levels of the first three LDDs dropped by 54–65% compared to the previous months and LDD 5 was lower than LDD 1–4 (*p* <  = 0.01 for all comparisons). In August, we observed the lowest ACh levels throughout larval development in the season. Median ACh levels declined by 63–79% on LDD 1–4 compared to April, May and June. Only LDD 3 and 5 were different from each other (*p* < 0.01). In September, the ACh levels rebounded close to July levels. LDD 1–3 were not different, LDD 4 had significantly less ACh than LDD 2 (*p* = 0.017), and LDD 5 significantly less than LDD 1–3 (*p* < 0.05 for all comparisons).

Similarly, the larval development day significantly influenced choline levels in all months from April to September (respective Chi-square(4) and significance values: 13.23, *p* = 0.01; 21.77, *p* < 0.001; 40.77, *p* < 0.001; 15.16, *p* < 0.01; 27.48, *p* < 0.001; 34.64, *p* < 0.001). As shown in Fig. 3 and Table 3, LDD 5 in April had higher choline levels than LDDs 1 and 2 (*p* < 0.05 for both comparisons). In May, LDD 4 and 5 were higher than LDD 3 (*p* < 0.01 for both comparisons). In June and September, we identified higher levels on LDD 5 compared to all other LDDs (June: *p* < 0.01 for all comparisons; September: *p* < 0.001 for all comparisons). July was the only month with higher choline than ACh levels throughout the entire larval development, LDD 5 showing higher levels than LDD 1 and 2 (*p* < 0.01 and *p* < 0.05, respectively). In August, choline levels on LDD 5 were higher than LDDs 2–4 (*p* < 0.001 for all comparisons).

### ACh and choline seasonality is robust across years and colonies.

We provide the variations in ACh and choline levels across LDDs and sampling months for the years 2020 to 2022 in Supplementary Figure S2 to assess the temporal variability of brood food composition, the seasonal patterns and year-to-year fluctuations. Given the variability across years, as well as colony-specific factors (e.g. size, genetics, and brood area), we utilized a generalized linear mixed model (GLMM) to analyse the data. The GLMM incorporated colony and year as random factors, and LDD and month as fixed factors. The corrected GLMM revealed significant results for ACh (*F*(29,1222) = 32.56, *p* < 0.001), indicating a high dependency on LDD (*F*(4,1222) = 82.95, *p* < 0.001), sampling month (*F*(5,1222) = 67.95, *p* < 0.001), and the LDD-month interaction (*F*(20,1222) = 5.97, *p* < 0.001). Similarly, choline levels showed significance in the corrected GLMM (*F*(29,1238) = 10.21, *p* < 0.001), with dependence on LDD (*F*(4,1238) = 38.44, *p* < 0.001), sampling month (*F*(5,1238) = 17.72, *p* < 0.001), and the LDD-month interaction (*F*(20,1238) = 2.27, *p* = 0.001). This highlights the robustness of our data across various years and colonies, affirming its reliability despite potential differences in colony performance and environmental conditions.

### Influence of abiotic factors

**Figure 4:**
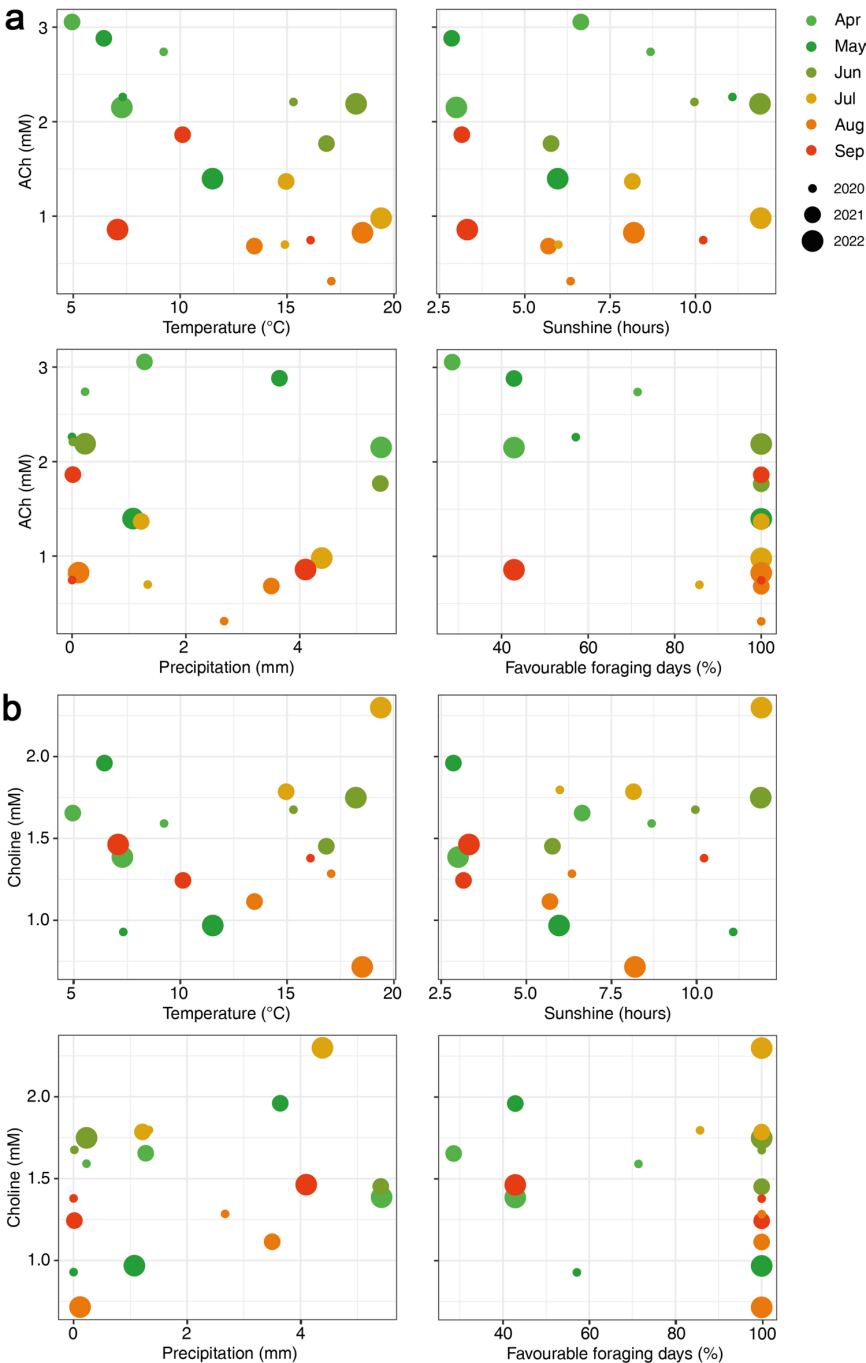
Influence of abiotic factors on ACh (a) and choline (b) levels. Scatterplots display the correlation of Temperature (°C), Sunshine (h), Precipitation (mm), and Favourable foraging days (%) with the mean ACh or choline level (mM) on each sampling day. The months are color-coded, and the size represents the year. Weather data from the nearest DWD weather station 2601 were averaged over the seven days preceding the sampling date. Favourable foraging days were determined as the number of days with a daily mean temperature above 10°C at our location (adjusted by + 3 °C for the ~ 550 m altitude difference to station 2601).

We investigated the impact of abiotic factors on our data, as they are expected to be linked to the available floral resources and the colony’s foraging intake. Research indicates that temperature and luminosity are the primary abiotic factors influencing honey bee foraging, with relative humidity, wind speed, and daytime precipitation having less significant effects^69,70^. In Fig. 4, we explored whether mean temperature, sunshine hours, favourable foraging days (mean temperature > 10 °C, review: ^71^) and precipitation in the seven days preceding the sampling dates impacted median choline and ACh levels per month and LDD (data from the closest Deutscher Wetterdienst weather station 2601; file “tageswerte_KL_02601_19360101_20221231_hist.zip” from https://opendata.dwd.de/climate_environment/CDC/observations_germany/climate/daily/kl/historical/; sunshine hours = direct solar irradiance exceeding 120 W/m^2^).

We found no discernible patterns or correlations between abiotic factors and ACh or choline levels. The color-coded dots representing early (green) and late (red) season in Fig.4 were scattered across the same x- or y-axis on multiple occasions. For instance, in 2022, low temperatures in September (7.1 °C) yielded a mean ACh level of 0.86 mM throughout larval development, whereas in April (7.3 °C), the level was 2.15 mM. Similarly, levels in July and August (~ 19 °C) were low, ranging from 0.83 to 0.86 mM like in September (7.1 °C). Similar irregularities were observed for the other factors. Therefore, we conclude that these factors alone do not significantly influence choline and ACh levels, such as via foraging activity. A comprehensive multi-year study, encompassing abiotic factors, foraging behaviour, floral abundance, and diversity, would be necessary to explore such correlations.

## Discussion

In this study, we show for the first time larval age- and season-dependent variation of choline and ACh concentrations in worker brood food across five larval development days, measured over six months and three years.

### Choline and ACh levels

Choline, classified as a lipogenic growth factor crucial for insect development, particularly in phospholipids, such as lecithins, exhibits unique efficacy in promoting larval growth, pupal development, and essential reproductive functions^72^. The significance of lipogenic growth factors is highlighted by the inability of caged honey bee colonies to rear brood beyond the age of 3–4 days without them, as observed by feeding diets containing only proteins, carbohydrates, lipids, vitamins, and minerals. Supplementing the diet with the lipogenic growth factor inositol (4.5 mg final concentration) supported larval development to the adult stage^73^. Therefore, the rise in choline levels in worker brood food when cell capping approaches observed in our study is presumed to be vital for LDD 4 and 5 larvae in sustaining the necessary quantity for forming cellular membranes. It is improbable that the observed increase is solely due to the hydrolysis of ACh in the brood food as the choline/ACh ratio rises from approximately 60% to 400% during development, suggesting a mechanism independent of ACh. The variation of choline levels throughout the season suggests a seasonal effect, possibly related to an additional role for this molecule. This may encompass the initiation of winter bee generation in August, marked by a reduction in brood area size and a cessation in offspring production across Germany. Winter bees seem to exhibit low levels of choline and elevated levels of O-phosphocholine, an intermediate in the synthesis of phosphatidylcholine, in comparison to summer bees^74^. Interestingly, choline levels in April 2018 (Fig. 1) exhibited inconsistencies compared to our data from 2020 to 2022, lacking the typical rise in late larval stages. The reason for this discrepancy remains uncertain; it could be an outlier related to sampling only one month of the year.

While ACh levels varied across seasons, they showed consistent differences in LLDs 4 and 5 independent of the season, revealing a decline in ACh levels towards cell capping. This trend aligns with a previous publication^49^ measuring ACh concentrations in worker brood food of 8.3, 5.5, and 1.2 mM for LDD 1–2, LDD 3, and LDD 4–5, respectively (categorized as below 5 mg, between 10 and 20 mg, and "older larvae" receiving modified worker jelly, starting on LDD 4 as described in the literature^34,75^). Despite some differences in mean concentrations, our study's comprehensive dataset, incorporating several hundred measurements per larval development day, highlights the reliability of these results. Additionally, our reliance on HPLC analysis contributes to increased precision compared to the pharmacological methodology used in the earlier study (eserine-treated rectus abdominis muscle of the frog and the small intestine of the albino mouse)^49^. We expect hydrolysis during storage to have no to negligible effects on our data and statistics, as we present consistent results in Supplementary Figure S2, independent of storage durations shown in Table 1.

### Possible relation in shift of brood food

Worker brood food can have a milky-white, watery-clear or yellowish appearance^76^. Our worker brood food samples displayed a milky-white appearance from LDD 1–3, transitioning to clearer and occasionally yellow samples in 4-day-old larvae (personal observation). At LDD 5, the food was scant, causing thickness that impeded capillary absorption. This aligns with description of worker brood food in literature, indicating age, caste, and season-dependent variations^34,77^. Jung-Hoffmann (1966) demonstrated a seasonal effect, with the white component comprising 20%, 27%, and 18% in June, and 25%, 39%, and 14% in August, on LDD 1–3, respectively. She proposed that the white component is likely a combination of mandibular and hypopharyngeal gland secretions, while the clear component involves hypopharyngeal gland secretions and honey. Furthermore, protein analyses indicated less hypopharyngeal gland secretion in the white component^34^. Considering ACh synthesis by membrane-bound ChAT in the hypopharyngeal glands^51^, we would anticipate higher ACh levels in the clear component, which is consistent with our data and Jung-Hoffmann’s findings. However, this pattern does not hold for the clear component in modified worker jelly, where ACh levels significantly dropped, suggesting a distinction between the clear components. Moreover, both existing literature and our own data (Supplementary Fig. S3) suggest that royal jelly has a lower ratio of the clear component compared to worker jelly^34^, yet it appears to exhibit higher ACh concentrations^49,51^. The observation that the clear component turns opaque if stored at room temperature^34^ implies additional complexity, highlighting the need for further research in honey bee larval nutrition.

### Influence of season and environmental factors

Our study revealed peak ACh concentrations from April to June and lowest levels in August, while choline peaked in July and dipped in August (Fig. 3). Despite year-to-year fluctuations (Supplementary Fig. S2), we confirmed significant dependencies of ACh and choline levels on LDD and season using a GLMM.

Our standard beekeeping practices included the extraction of honey, *Varroa destructor* treatment, and feeding with 72.7% sugar solution (Apiinvert, Südzucker, Mannheim; either one-time 5 kg, or 3 kg followed by another 3 kg 10 days later) to prepare colonies for winter, between mid-August and mid-September. The colonies were not fed pollen supplements. In June 2021, we exceptionally fed approximately 4 kg of Apiinvert, as colonies had no nectar inflow due to long periods of unfavorable rainy weather, which were typical for that year. In contrast, 2022 was exceptionally hot and dry, and from mid-July to mid-September, colonies exhibited significantly reduced breeding activity for about six weeks, with a lack of pollen in the hives due to drought. These events correspond with reduced sample sizes, as shown in Table 1. We ruled out *Varroa destructor* treatment effects, noting consistent low levels in August despite treatment initiation in early September 2022.

Honeybees exhibit seasonal behavioural adaptations, suggesting colony awareness of seasonal changes. Factors such as abiotic conditions and nutrition likely influence worker responses and physiology, impacting aspects like winter bee generation ^48^. However, when correlating key foraging abiotic factors like temperature and sunshine hours with ACh and choline levels, we found no significant associations, suggesting that seasonal variations may rather be linked to changes in resource availability and nutritional quality.

Numerous studies have demonstrated seasonal dynamics in pollen quality, quantity and composition, impacting honey bee colony and individual development^12–17,78–80^, with only little contrary evidence^81^. These investigations reveal that bee-collected pollen protein peaks in July and decreases through September^78–80^, and that spring pollen has higher protein^13^ or gland-stimulating amino and lipid acids^16,17^ than autumn pollen. This corresponds with our observed low levels of ACh and choline in the same timeframe and is potentially directly correlated, suggesting a decrease in ACh and choline with declining pollen protein and quality. Furthermore supply–demand deficits during August/September could be the cause, as it has been shown for bumble bee colonies in the UK, suggesting inadequate supply to meet demand^82^.

Significantly, the nutritional landscape for honey bee colonies is contingent on their geographical location, influenced by agricultural land-use practices, thereby affecting pollen and protein dynamics^78,79,83^. For example, honey bee health tends to be compromised in July in agricultural regions, coinciding with the mass-flowering of maize (*Zea mays L.*) that produces poor-quality pollen deficient in the essential amino acid histidine^14^. Similar effects might be present in our study, induced by regional vegetation.

## Conclusion

Our study highlights the consistent influence of larval development days and sampling month on choline and ACh levels across multiple years and colonies. Future investigations should engage colonies subjected to diverse pollen availability and quality to validate the seasonal influence of pollen on ACh levels in worker brood food among *Apis mellifera* colonies. Furthermore, exploring the physiological repercussions of ACh variations on worker bees in such contexts is essential and could provide insights into winter bee generation. Understanding the mechanism of ACh in larval food and its role in development, including receptor types, locations, and functions, will deepen our understanding of honeybee colonies and potentially other social insect colonies. We should explore which other social insect species utilize ACh for brood rearing, like during adult-larvae trophallaxis of ants^84^, and the evolutionary advantage it provides.

### Supplementary Information

Supplementary Figures.

## Data Availability

Data are available from the publicly accessible digital repository OSF: https://osf.io/3m795/?view_only=34c94a69e7054af590c6223d6f0c358b .
