# Supplementary material for: Acetylcholine and choline in honey bee (Apis mellifera) worker brood food are seasonal and age-dependent
Source: Sci Rep. 2024 Aug 6;14:18274. doi: 10.1038/s41598-024-68650-x (PMC11303543; doi:10.1038/s41598-024-68650-x)
Supplement: Supplementary file 1 — Supplementary Figures. [file 41598_2024_68650_MOESM1_ESM.pdf]

Acetylcholine and choline in honey bee (*Apis mellifera*) worker brood food are seasonal and age-dependent – Supplementary material

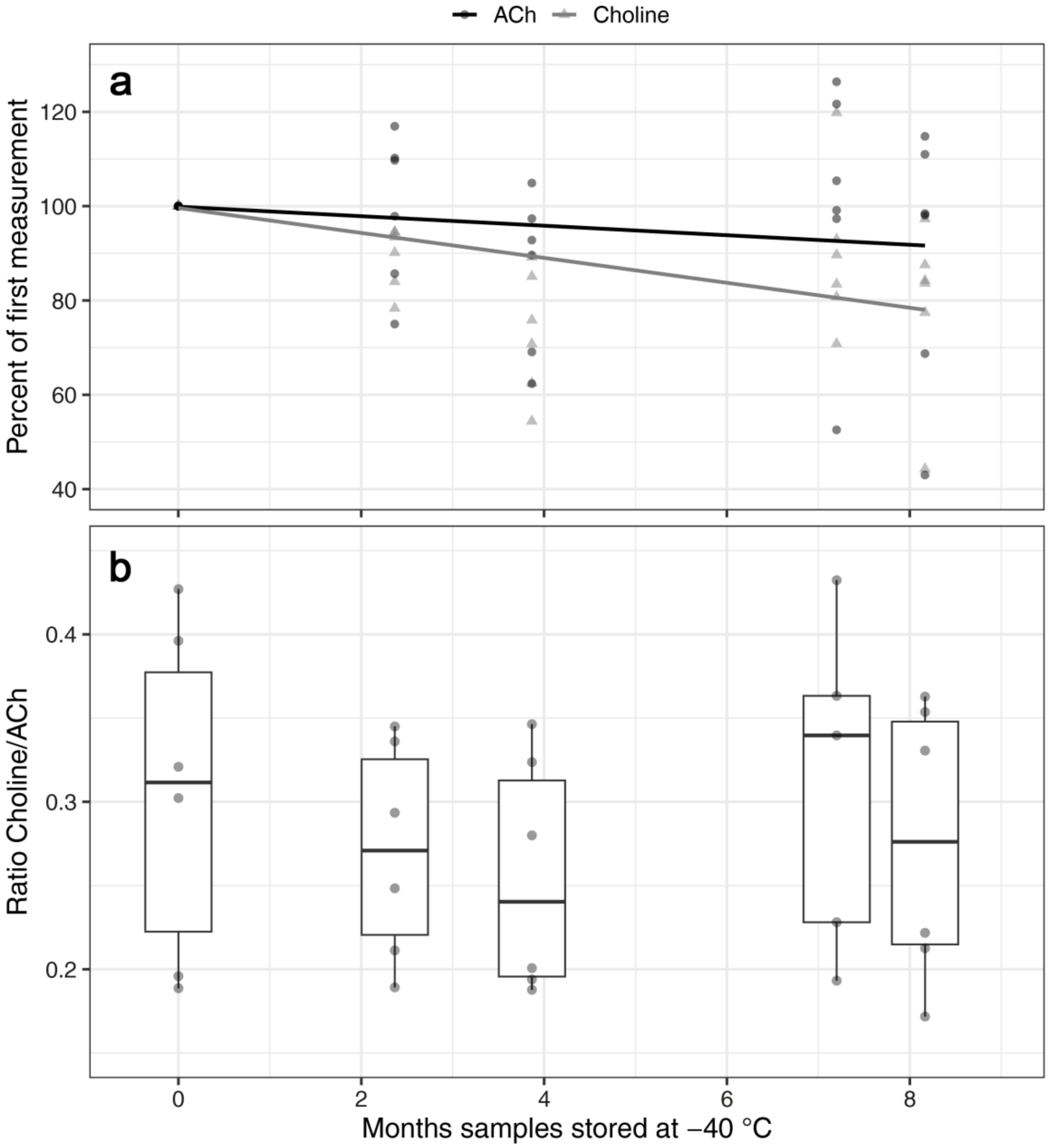

Supplementary Figure S1: Stability analysis of choline and ACh over 8-month storage at -40°C. Worker brood food aliquots, collected in late March 2022 from cells with ~3-day old larvae, were stored at -40°C. Every two months, 6 aliquots were analysed over an 8-month period. Percentage change in concentration (**a**) and choline/ACh ratio (**b**) were assessed to anticipate ACh hydrolysis. Initial ACh concentration remained at 97.5%, 96.0%, 92.6%, and 91.6% after 2.4, 3.9, 7.2, and 8.1 months, respectively, while choline levels were at 93.4%, 89.4%, 80.6%, and 80.6%. Despite expectations, no statistical difference in the median choline/ACh ratio was observed over time, suggesting simultaneous degradation of ACh and choline. Overall, ACh showed minimal hydrolysis, maintaining approximately 92% of its original concentration, while choline levels decreased to 81% over 8 months.

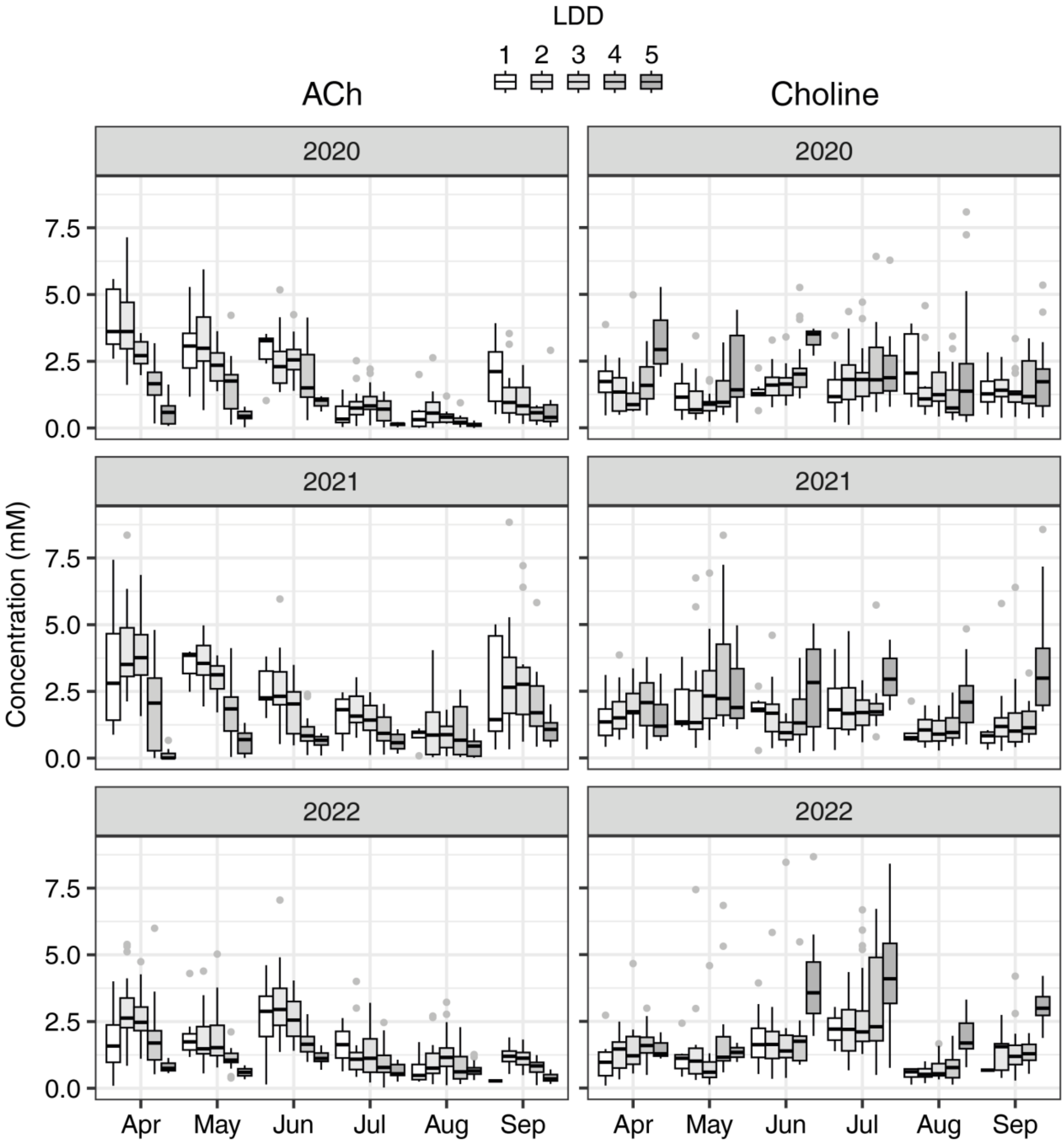

Supplementary Figure S2: Yearly variations in worker brood food ACh and choline levels per larval development day (LDD) and month (Apr–Sep). Each subplot within the figures represents a specific year, allowing for a visual examination of the yearly trends in ACh and choline concentrations.

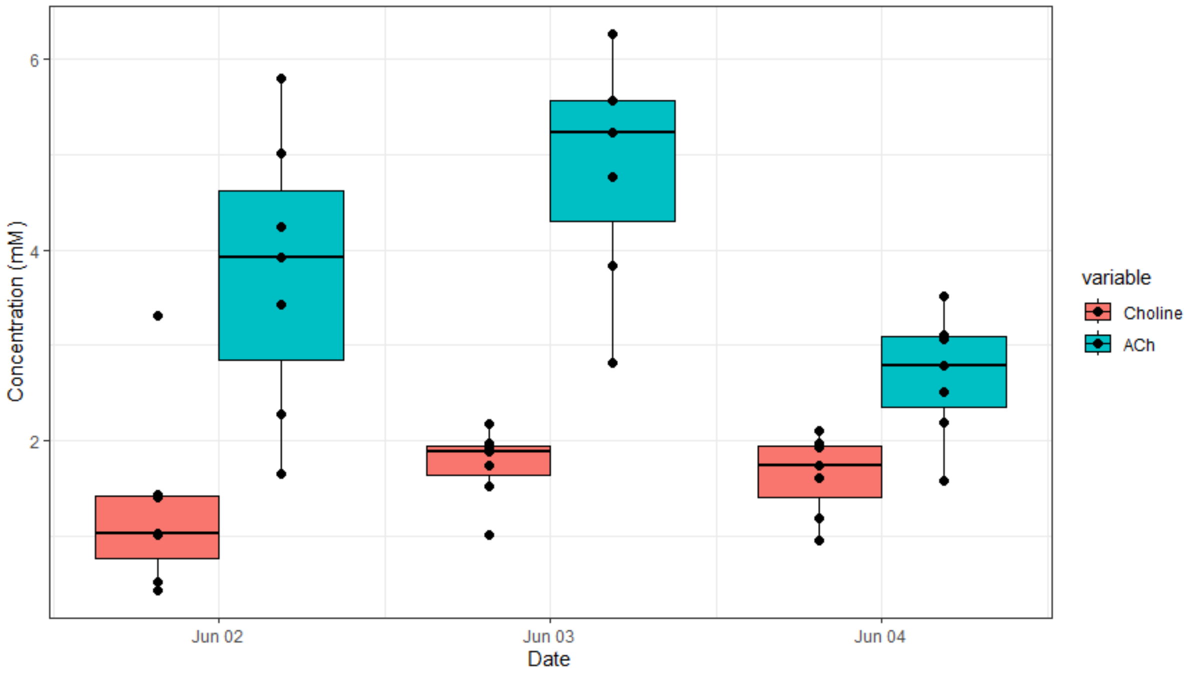

Supplementary Figure S3: Choline and ACh concentrations (mM) in royal jelly. From June 2nd to June 4th 2020, we collected daily royal jelly samples from seven queen cells using the analysis method described in the manuscript. These queen cells were obtained from our local queen rearing program. While we did not confirm larval development by weight or time, all cells were capped on June 5th, suggesting that the samples represented queens 1–3 days before capping.
